# Efficient control of Japanese encephalitis virus in the central nervous system of infected pigs occurs in the absence of a pronounced inflammatory immune response

**DOI:** 10.1186/s12974-020-01974-3

**Published:** 2020-10-23

**Authors:** Valerie Redant, Herman W. Favoreel, Kai Dallmeier, Willem Van Campe, Nick De Regge

**Affiliations:** 1 Operational Direction Infectious Diseases in Animals, Unit of Enzootic, Vector-borne and Bee Diseases, Sciensano, Groeselenberg 99, 1180 Brussels, Belgium; 2 Department of Virology, Immunology and Parasitology, Faculty of Veterinary Medicine, Ghent University, Salisburylaan 133, 9820 Merelbeke, Belgium; 3 Rega Institute for Medical Research, Department of Microbiology & Immunology, KU Leuven, Herestraat 49, 3000 Leuven, Belgium; 4 Experimental Animal Center, Sciensano, Kerklaan 68, 1830 Machelen, Belgium

**Keywords:** Japanese encephalitis virus, Pig, Virus dissemination, Immune response

## Abstract

**Background:**

Japanese encephalitis virus (JEV) is the leading cause of viral encephalitis in Asia. JEV infection of mice and humans can lead to an uncontrolled inflammatory response in the central nervous system (CNS), resulting in a detrimental outcome. Pigs act as important amplification and reservoir hosts, and JEV infection of pigs is mostly subclinical. Information on virus spread in the CNS and immune responses controlling JEV infection in the CNS of pigs, however remains scarce.

**Methods:**

Nine-week-old pigs were inoculated intranasal or intradermal with a relevant dose of 10^5^ TCID_50_ of JEV genotype 3 Nakayama strain. Clinical signs were assessed daily, and viral spread was followed by RT-qPCR. mRNA expression profiles were determined to study immune responses in the CNS.

**Results:**

Besides a delay of 2 days to reach the peak viremia upon intranasal compared to intradermal inoculation, the overall virus spread via both inoculation routes was highly similar. JEV appearance in lymphoid and visceral organs was in line with a blood-borne JEV dissemination. JEV showed a particular tropism to the CNS but without the induction of neurological signs. JEV entry in the CNS probably occurred via different hematogenous and neuronal pathways, but replication in the brain was mostly efficiently suppressed and associated with a type I IFN-independent activation of OAS1 expression. In the olfactory bulb and thalamus, where JEV replication was not completely controlled by this mechanism, a short but strong induction of chemokine gene expression was detected. An increased IFNy expression was simultaneously observed, probably originating from infiltrating T cells, correlating with a fast suppression of JEV replication. The chemokine response was however not associated with the induction of a strong inflammatory response, nor was an induction of the NLRP3 inflammasome observed.

**Conclusions:**

These findings indicate that an adequate antiviral response and an attenuated inflammatory response contribute to a favorable outcome of JEV infection in pigs and help to explain the limited neurological disease compared to other hosts. We show that the NLRP3 inflammasome, a key mediator of neurologic disease in mice, is not upregulated in pigs, further supporting its important role in JEV infections.

## Background

Mosquito-borne flaviviruses represent a major group of neurotropic viruses, including dengue virus, yellow fever virus, West Nile virus (WNV), and Japanese encephalitis virus (JEV) [1]. JEV is a zoonotic virus that is currently endemic in rural areas of South and Southeast Asia. It is mainly transmitted by *culex* mosquitoes to pigs and water birds, which act as important amplification and reservoir host. Transmission to humans, horses, and cattle by mosquitoes can occur but due to low viremia, these function as a dead-end hosts. In Asia, JEV is the leading cause of viral encephalitis in humans. Mostly, the virus causes a subclinical infection. However, in 1 in 250 cases, JEV can lead to severe clinical disease. These cases are characterized by high fever, headache, coma, and convulsions. Mortality associated with encephalitis caused by JEV is as high as 25–30% of all cases with severe clinical disease, and around 50% of all surviving patients will suffer neurological sequelae [2–4].

In contrast, symptoms in pigs are typically mild ranging from fever, reduced appetite, and reluctance to move. Young piglets can develop non-suppurative encephalitis while infection of JEV in mature pigs primarily manifests as a reproductive disease with abortions, stillbirths, and transient infertility [5]. The natural infection rate in pigs reaches 98 to 100%, and infection is associated with a high viremia which lasts for 2–4 days, making that pigs represent an ideal amplification host for further spread by vector mosquitoes [1]. Interestingly, it was recently shown that also a direct vector-free transmission of JEV between pigs can occur, further emphasizing the important role of pigs in JEV epidemiology and spread [6].

Most research on JEV pathogenesis has focused on humans and mice, while pathogenesis in pigs, one of its natural hosts, remains poorly studied. Only recently, a more detailed pathogenesis study in pigs has been conducted and showed that JEV has a clear tropism for tonsils and neurological tissues after intravenous inoculation with a high dose (10^7^ TCID_50_) of JEV. The study also indicated that the virus persists in the tonsils till at least 25 dpi [7]. Another study conducted in piglets shows that severe brain lesions are induced by JEV upon intranasal inoculation with a dose of 10^6^ TCID_50_ [8]. Despite these studies, it remains unclear as to how JEV spreads throughout the body, how it enters the central nervous system (CNS), and whether these aspects differ depending on the inoculation route. Also the immune response controlling JEV infection in the CNS of pigs remains largely unstudied. While studies in mice and humans have shown that JEV infection in the CNS is accompanied by an extensive ongoing inflammation and increased neuronal cell death, thereby contributing to a deleterious outcome of a JEV infection [9–14], this might be different in pigs where the course of infection usually remains subclinical. To study all these aspects in detail, we performed an extensive in vivo infection experiment to compare JEV spread and replication and the associated immune response in pigs after inoculation with a relevant low dose of JEV via the oronasal route, mimicking the vector-free transmission, or via the intradermal route, mimicking natural infection by the mosquito.

## Methods

### Virus

JEV genotype 3 Nakayama strain was purchased from Culture Collections (UK). The virus was passaged once in Vero cells before use in the animal experiment. This resulted in a JEV virus stock with a titer of 10^6^ TCID_50_/ml. The virus was then passaged another two times in order to have a sufficient stock for downstream laboratory analyses.

### Animals and experimental design

The procedures executed in this experiment were approved by the ethical committee of Sciensano (20171024-01) and conducted in BSL3 animal facilities. All 59 pigs (Belgian landrace sows crossbred with Piétrain boars) were 8 weeks old when they were brought into the BSL3 facilities and were allowed 1 week of adaptation to their new environment before the start of the experiment. The animals were divided randomly into 3 groups. The first group of 24 pigs was inoculated intranasally with a final infectious dose of 10^5^ TCID_50_ of JEV Nakayama strain per animal by using a nozzle mounted syringe. The second group of 24 pigs was inoculated intradermally in the neck by the use of a syringe with a final dose of 10^5^ TCID_50_ of JEV Nakayama strain per animal. The third group of 11 pigs was mock-infected and kept as a control group.

Clinical signs were assessed daily using a scoring card whereby each animal was scored for general condition, respiratory signs, appetite, and neurological symptoms. For each of these groups, a score of 0 indicated no nasal discharge, no coughing, normal breathing, normal appetite, and no neurological symptoms, respectively. A score of 1 is given for mild mucous discharge, spontaneous coughing (1–3/5 min), increased respiration rate, reduced appetite, depression, panting, and nibbling. A score of 2 is given for marked mucous discharge, coughing (3/5 min), abdominal breathing, lethargy, no appetite, tremor, circling, scratching, and ataxia. Clinical signs such as purulent nasal discharge, gasping, convulsions, and paralysis were scored as 3. Also rectal temperatures were monitored. Three pigs from the control group were euthanized on the day of infection (0 dpi). At 1, 2, 3, 5, 7, 10, 14, and 21 dpi, one predefined pig of the control group and three predefined pigs of the intranasal and intradermal inoculated groups were euthanized. At the time of euthanasia, the blood (for serum and leukocyte pellets), nasal swabs, and multiple tissues were collected (prescapular lymph node, spleen, liver, kidney, cerebrum, cerebellum, thalamus, brain stem, olfactory bulb, and trigeminal ganglia). Per time point, the blood (for serum) and nasal swabs were collected from another three predefined pigs per group. Skin biopsies at the site of inoculation were collected only from pigs of the intradermal inoculated group till 7 dpi.

### JEV detection by qPCR

Except for lymph nodes and skin samples, around 0.5 cm^3^ of tissue was homogenized in 1 ml of phosphate-buffered saline (PBS) with 2 zirconium beads by high-speed shaking (4 min, 25 Hz) in a TissueLyser (Qiagen). Lymph nodes were homogenized in 1 ml of PBS with 2 5 mm stainless steel beads (Qiagen) and high-speed shaking (4 min, 25 Hz) in a TissueLyser. Nasal swabs were submerged in 1 ml PBS and vortexed for 30 min. Leukocyte pellets were prepared from whole blood samples collected at the moment of euthanasia. Two milliliters of the blood was added to 8 ml of hemolysis buffer. After 20 min of incubation at room temperature and subsequent centrifugation, the leukocyte pellet was obtained and homogenized using the QiaShredder kit (Qiagen). RNA was extracted from serum, pretreated nasal swabs, and homogenized tissues and cells by the use of the RNeasy Mini kit (Qiagen) according to the manufacturer’s instructions.

RNA extraction from the skin samples was done as follows. The frozen skin was immediately submerged in 1 ml of TRIzol and homogenized using the ULTRA-TURRAX. After addition of 200 μl chloroform and centrifugation at 14 000 rpm for 15 min, the aqueous layer containing the RNA was collected. This was further processed using the RNeasy Mini kit according to the manufacturer’s instructions.

A qPCR amplifying the 3′ NTR region of JEV [15] was used to detect the presence of JEV RNA in the RNA extracts of different tissues. The AgPath-ID one-step RT-PCR kit was used (Thermo Fisher) for amplification with a final primer and probe concentration of 0.4 μM and 0.25 μM, respectively. The RT-qPCRs were run on a LightCycler 480 (Roche) for 45 cycles. A standard curve was constructed by testing a 10-fold serial dilution of a JEV virus stock with a titer of 10^8.5^TCID_50_/ml in the qPCR described above, and used to convert Ct values in equivalent viral loads in TCID_50_/ml or TCID_50_/g.

### JEV isolation and titration in serum, swabs, and tissue samples

The presence and quantification of infectious virus in the serum, nasal swabs, and tissues was determined as follows. First, homogenates were prepared as described above for JEV detection by qPCR. Thereafter, homogenates of the serum, nasal swabs, olfactory bulb, thalamus, cerebrum, cerebellum, and lymph nodes were serially diluted till 10^-3^. One hundred microliters of all dilutions was then added in quadruplicates to 90% confluent Vero cells grown in 96-well plates. After 2 h of incubation, the inoculum was removed, and cells where washed and replaced by fresh DMEM medium and incubated at 37 °C. After 72 h of incubation, the plates were put at − 80 °C and freeze-thawed. A second passage was then conducted by transferring 100 μl of supernatants of the first passage to 90% confluent Vero cells. This was incubated for 2 h at 37 °C before replacing the inoculum with fresh DMEM medium. After 72 h of incubation at 37 °C, supernatants were removed and the cell layer was fixed and permeabilized by the addition of methanol at − 20 °C for 20 min. Cells were subsequently stained with the primary flavivirus 3571 antibodies (Santa Cruz), followed by incubation with a FITC-labeled secondary antibody to visualize JEV replication under the fluorescence microscope.

### Virus neutralization test

The presence of neutralizing antibodies was assessed by virus neutralization tests (VNT). Sera were 1:5 diluted in DMEM, followed by two-fold dilutions till 1:640 in 96 well plates in final volumes of 50 μl. One hundred TCID_50_ of the virus in 50 μl was added to the serum dilutions and incubated for 1 h at 37 °C. 10^4^ Vero cells in 100 μl medium were then added to the serum-virus mix and incubated for 6 days at 37 °C. The presence of a cytopathic effect was assessed under the light microscope to check for virus replication. All sera were tested in duplicate. The neutralizing titer of a sample was determined as the highest dilution of the serum that was still capable to completely neutralize the virus.

### cDNA synthesis and preamplification

Following the manufacturer’s instructions, the extracted RNA was treated with Turbo DNase (Thermo Fisher) to eliminate contaminating genomic DNA. The DNase-treated RNA samples were subsequently converted to cDNA using the M-MLV reverse transcriptase system (Life Technologies). For each reaction, a mix of 4 μl 5 × first-strand buffer, 2 μl 0.1 M DTT, 1 μl 10 nM dNTP mix, 0.2 μl 10 × hexanucleotide mix, 0.5 μl M-MLV RT, 8.3 μl H_2_O, and 4 μl RNA was prepared and incubated at 37 °C for 45 min. This was followed by inactivation at 95 °C for 10 min. In order to increase the available amount of cDNA, a preamplification step was carried out using the TaqMan PreAmp master mix (Applied Biosystems) following the manufacturer’s instructions. The mix of primers used to preamplify the obtained cDNA consisted of a selection of reference genes and targets of interest (Additional file 1). The preamplification program was as follows: denaturation at 95 °C for 10 min and 14 cycles of amplification 15 s at 95 °C and 4 min at 60 °C. The preamplification uniformity was verified by testing one selected sample of all tissues (thalamus, olfactory bulb, cerebrum, and cerebellum) for all reference genes and target genes before and after preamplification, and PreAmp uniformity was calculated per reference gene (ACTB, GADPH, PPIA, UBC). Normalized Ct values were calculated for the different target genes before (cDNA) and after preamplification (PreAmp) according to the following formulas: ΔCT[cDNA] = CT[cDNA target gene] − CT[cDNA reference gene] and ΔCT[PreAmp] = CT[PreAmp target gene] − CT[PreAmp reference gene], respectively. The ΔΔCT was subsequently calculated from the difference of the two ΔCTs: ΔΔCT = ΔCT[PreAmp] − ΔCT[cDNA]. An ideal PreAmp uniformity is reached when ΔΔCT values lay between − 1.5 and + 1.5. The mean PreAmp uniformity values for the target genes in the thalamus related to the reference genes ACTB, GADPH, PPIA, and UBC were − 1.30, − 0.31, 0.18, and − 0.10, respectively. The mean PreAmp uniformity values for the target genes in the olfactory bulb related to the reference genes ACTB, GADPH, PPIA, and UBC were − 0.91, − 0.13, 0.50, and 0.22, respectively. The mean PreAmp uniformity values for the target genes in the cerebrum related to the reference genes ACTB, GADPH, PPIA, and UBC were − 1.03, − 0.59, 0.82, and − 0.40, respectively. The mean PreAmp uniformity values for the target genes in the cerebellum related to the reference genes ACTB, GADPH, PPIA, and UBC were − 1.20, − 0.55, 0.29, and − 0.34, respectively.

### mRNA expression by qPCR and relative quantification

Primers and probes for 4 reference genes (ACTB, GADPH, PPIA, and UBC) and 7 targets of interest (IFNα, IFNβ, IFNγ, TNFα, IL1α, IL6, and IL10) were designed during previous research [16]. Primers and probes for 10 other targets of interest (IL1β, IL18, CCL2 (MCP1), CCL5 (RANTES), CXCL9, CXCL10 (IP10), CXCL11, NLRP3, CASP1, OAS1) were designed using Primer3 based on porcine sequences available in NCBI databases and purchased from Integrated DNA Technologies (IDT). The specificity of the primers was verified in BLAST, and a classic PCR was run for each primer pair to reassure the correct size of the amplicon. All used sequences are shown in Additional file 1.

If possible, a duplex qPCR was performed using 6-carboxyfluorescein (FAM) and Hexachloro-fluorescein (HEX)-labeled probes. Each qPCR reaction consisted out of 5 μl preamplified sample, 10 μl of 2× FastStart TaqMan Probe master mix (Roche), 1 μl primer/probe mix (final concentration of 0.5 μM and 0.25 μM, respectively), and 3 or 4 μl of H_2_O (duplex or monoplex reaction, respectively) with a final volume of 20 μl. The following qPCR program was run: polymerase activation and denaturation at 95 °C for 10 min, followed by 45 cycles of denaturation at 95 °C for 15 s and annealing/extension at 60 °C for 45 s. All samples were run in duplicate on a LightCycler 480 real-time PCR system (Roche).

ACTB, GADPH, PPIA, and UBC were used in qbase plus as a means of normalization for CNS samples, as previously determined [16]. The quantification and normalization of the expression levels were based on the calculation of target *C*_*T*_ values and reference gene *C*_*T*_ values in qBasePlus software. The expression levels were normalized with respect to the selected reference genes and to technical and experimental errors. Relative expression quantification analysis relied on the qBasePlus method [17]. Individual target gene levels for all animals were expressed relative to the average of the control group.

### Histopathology and immunohistochemistry

Formalin-fixed and preserved cerebrum samples were embedded in paraffin, cut at 5 μm, and stained with hematoxylin Gill III and eosin.

Immunohistochemistry (IHC) staining for IBA1 was performed on the same paraffin-embedded samples. The anti-goat HRP-DAB cell and tissue staining kit (R&D systems) was used according to the manufacturer’s instructions. In short, steamed antigen retrieval was executed in 0.05% Tween20 TRIS-EDTA buffer. Next, samples were treated with peroxidase, serum, avidin, and biotin block before incubation with the primary antibody. The primary IBA1 antibody (PA5-18039 from Thermo Fisher) was used at a 1:100 dilution in TRIS-EDTA buffer with 0.05% tween20 and incubated for 1 h at room temperature. Visualization was obtained using the HRP-DAB system included in the staining kit. Sections were counterstained with hematoxylin Gill III, mounted with DPX, and visualized with an Olympus light microscope. The staining intensity between the samples was quantified using the IHC profiler plugin for ImageJ. The plugin deconvolutes the image into hematoxylin or DAB and scores the intensity of the staining by calculating a contributing percentage of high positive, positive, low positive, and negative values. Based on the contributions of the different groups, a score (negative, low positive, positive, high positive) is given to the sample [18].

Fluorescence IHC was performed on snap-frozen, methyl cellulose-embedded cerebrum samples. Frozen sections were cut at 10 μm in duplicate per animal and dried overnight before fixation in acetone at − 20 °C. After rehydration, sections were blocked for 1 h with 10% goat serum in TRIS buffer at room temperature. Cryosections were stained with primary antibody at a 1:100 dilution of mouse anti-porcine CD3 (clone PPT3, BioRad) in a 2% BSA TRIS buffer overnight at 4 °C. The following day, cryosections were stained with goat anti-mouse daylight 549 (Thermo Fisher) at a dilution of 1:200 for 1 h at room temperature. Staining was analyzed using a Leica fluorescent microscope, and quantification was performed by counting of CD3^+^ cells in 4 squares of 0.0875 mm^2^ per tissue in the LasX software.

### Statistical analysis

Statistical analysis was performed using GraphPad Prism. Kruskal-Wallis and Mann-Whitney tests were used to assess whether differences in median viral loads were present between different tissues or between animals within a specific tissue. Chi-square and Fisher’s exact tests were used to determine whether significant differences were present between the number of JEV-positive pigs between inoculation routes or between tissues within a specific inoculation route. *P* values < 0.05 were considered to be significant.

## Results

### Clinical symptoms

At the start of the experiment, all animals were healthy. During the experiment, the pigs did not show any signs of illness such as reluctance to move or reduced appetite. Also no neurological symptoms were observed. Considering that the normal body temperature of pigs lies between 38.5 and 40 °C, most pigs in the intranasal and intradermal inoculated group had a slight fever at 8 dpi with a mean body temperature of 40.4 and 40.2 °C for both groups, respectively (Additional file 2).

### Nasal JEV excretion

**Fig. 1:**
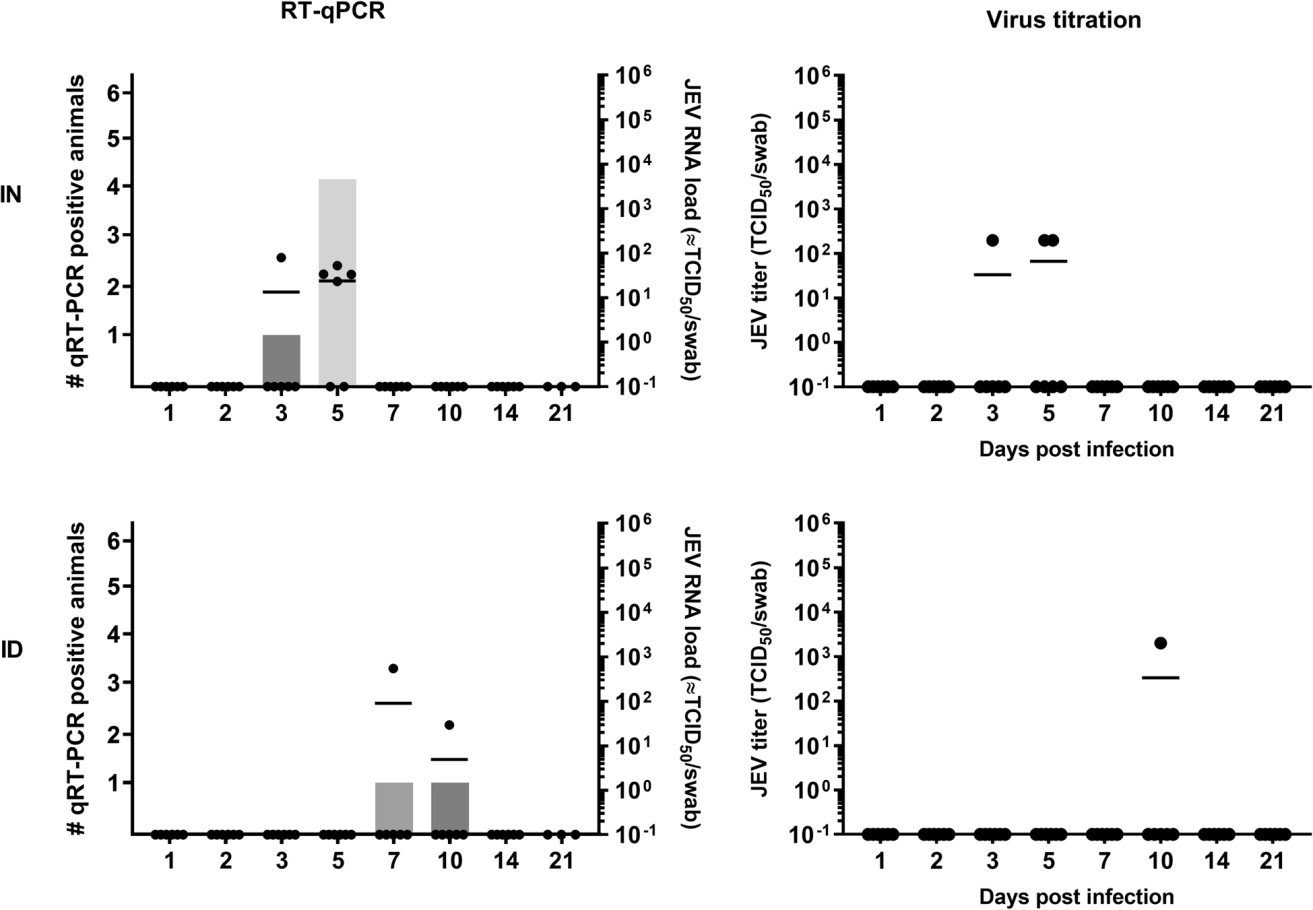
Nasal JEV excretion. Number of JEV excreting animals (bars) and JEV RNA loads (dots) in the nasal swabs (left panels) and amount of infectious JEV in swabs determined by virus titrations (right panels) upon intranasal (IN) and intradermal (ID) inoculation of 9-week-old pigs with 10^5^ TCID_50_/animal

Only a few animals tested RT-qPCR-positive for JEV in nasal swabs of both experimental groups (Fig. 1). Five out of 24 and 2 out of 24 animals were found JEV RNA-positive with high Ct values (indicating low amounts of viral RNA) in the intranasal and intradermal inoculated group, respectively. JEV RNA was found at 3 and 5 dpi after intranasal inoculation while this was only at 7 and 10 dpi upon intradermal inoculation. Virus isolations showed that some of the RT-qPCR positive samples (3 out of 5 after intranasal inoculation; 1 out of 2 after intradermal inoculation) also contained infectious virus, but with low viral loads between 10^2^ and 10^3^ TCID_50_/ml (Fig. 1).

### Initial JEV replication and dissemination upon inoculation

**Fig. 2:**
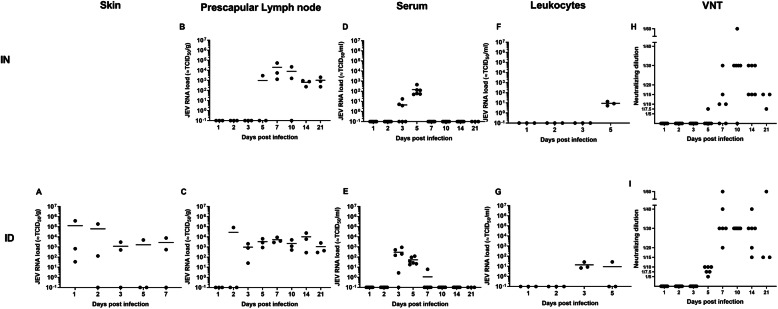
JEV dissemination. JEV RNA loads in the skin (**a**), prescapular lymph nodes (**b**, **c**), serum (**d**, **e**), and leukocyte pellets (**f**, **g**) measured by RT-qPCR upon intranasal (ID) and intradermal (ID) inoculation of 9-week-old pigs with 10^5^ TCID_50_/animal, in correlation with neutralizing antibody titers measured by virus neutralization tests (VNT) (**h**, **i**). Each dot represents one animal

Upon intradermal JEV inoculation in the neck region, JEV RNA was detectable in skin biopsies collected at the initial site of inoculation from 1 dpi onwards till 7 dpi (end of skin testing) in most pigs (Fig. 2a). Next, at 2 dpi, the draining prescapular lymph node of 1 out of 3 examined pigs became JEV RNA-positive. From 3 days post intradermal inoculation, all pigs were JEV RNA-positive in this lymph node till the end of the experiment at 21 dpi (Fig. 2c). The amount of viral JEV RNA seemed to be stable over time and equivalent to about 10^3^ à 10^4^ TCID50/g. No virus could be isolated from any of those lymph nodes. Next to the detection in draining lymph nodes at 2 dpi, JEV RNA was detected in the serum of 5 out of 6 pigs at 3 dpi. It immediately reached its peak titer at that time point, and the mean viral load was maintained at the same level in positive pigs till 5 dpi (Mann-Whitney; *P* = 0.662). By day 7 dpi, viral RNA could only be detected in low amounts in the serum of 1 out of 6 tested animals (Fig. 2e). Similar results were found by virus isolation with viral titers ranging between 10^2^ and 10^4^ TCID50/ml serum during viremia (Additional file 3). Coinciding with the detection of JEV RNA and infectious virus in the serum, JEV RNA could also be detected in the leukocyte pellets at 3 dpi. At 5 dpi, 1 out of 3 animals tested was positive for JEV RNA in leucocyte pellets (Fig. 2g).

Upon intranasal inoculation, JEV RNA was first detected at 3 dpi at low amounts in the serum of 3 out of 6 pigs. Viral titers then increased (Mann-Whitney; *P* = 0.024) and reached their peak at 5 dpi (Fig. 2d). These results were confirmed by virus isolation (Additional file 3). Also leukocyte pellets were positive for JEV RNA at the moment of peak viremia at 5 dpi (Fig. 2f). Interestingly, JEV RNA could also be detected in prescapular lymph nodes of intranasal infected pigs from the moment of peak viremia at 5 dpi onwards (Fig. 2b). Also here, no infectious virus could be detected via viral titration assays in prescapular lymph nodes.

Independent of the inoculation route, the almost complete drop in viremia by 7 dpi correlated with the detection and increase of neutralizing antibodies from 5 dpi onwards (Fig. 2h, i).

Coinciding with the peak of JEV RNA detection in the blood, JEV RNA was also detected in internal organs such as the liver and kidney (Additional file 4), but only in low amounts (equivalent to 10^2^–10^3^ TCID_50_/g tissue). JEV was completely eliminated from these organs after 10 to 14 dpi. JEV RNA was found in the spleen till the end of the experiment, but without indications for virus replication based on the stable JEV RNA load detected in RT-qPCR (Additional file 4).

### JEV entry into the CNS

**Fig. 3:**
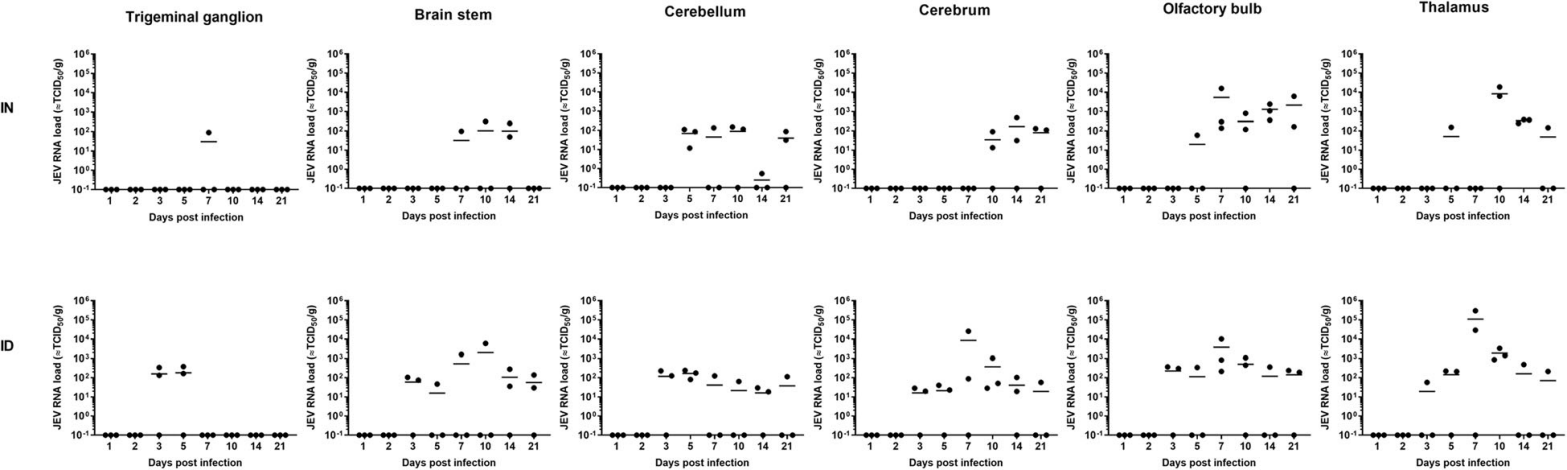
JEV tropism to the CNS. JEV RNA loads as detected by RT-qPCR in the trigeminal ganglion, brain stem, cerebellum, cerebrum, olfactory bulb, and thalamus upon intranasal (IN) and intradermal (ID) inoculation of 9-week-old pigs with 10^5^ TCID_50_/animal. Each dot represents one animal

JEV RNA was detected in all CNS parts that were examined (Fig. 3). The first appearance mostly coincided with the moment of the peak viremia, being at 5 dpi intranasal and 3 dpi intradermal inoculation. Only the spread to the trigeminal ganglion, brain stem, and cerebrum took a few days longer after intranasal inoculation. It was first analyzed whether there were differences in the efficiency of JEV spread to the different brain parts depending on the inoculation route. Since JEV reached the CNS later upon intranasal than intradermal inoculation, only pigs euthanized from the day of peak viremia till the end of the experiment were taken into account for this comparison (i.e., 3 till 21 dpi for intradermal inoculation and 5 till 21 dpi for intranasal inoculation). No difference was found in the total number of positive brain tissues (39/90 for intranasal vs 58/108 for intradermal inoculation; Fisher’s exact test; *P* = 0.16) and in the number of positive samples per brain part (*P* values > 0.05 for each Fisher’s exact test) between both inoculation routes.

Significant differences were however found when looking at the number of positive samples between the different brain parts for each inoculation route separately. Upon intranasal inoculation, the least positive samples were found in the trigeminal ganglion (1/15) and brain stem (4/15) and most positive samples in the thalamus (7/15), cerebellum (9/15), and olfactory bulb (11/15) (chi-square test; *P* = 0.004). Also after intradermal inoculation, significantly less positive samples were found in the trigeminal ganglion than in the olfactory bulb and cerebrum (Fisher’s exact tests; *P* = 0.02 and 0.04, respectively). Except for the trigeminal ganglion, a similar number of positive samples were found in the brain stem (9/18), cerebellum (10/18), thalamus (10/18), olfactory bulb (11/18), and cerebrum (12/18) after intradermal inoculation.

It was next analyzed by RT-qPCR whether differences were present in the overall viral load found in the different parts of the brain for each inoculation route. Seen the limited number of positive samples, the trigeminal ganglion and brainstem were excluded from the analysis after intranasal inoculation and the trigeminal ganglion from the intradermal inoculation. The median viral load in RNA-positive pigs significantly differed between brain parts after intranasal (Kruskal-Wallis; *P* = 0.004) and intradermal (Kruskal-Wallis; *P* < 0.001) inoculation. The highest median viral loads were found in the thalamus and olfactory bulb for both inoculation routes. Two-by-two comparisons via Mann-Whitney tests also showed that the median viral loads in these parts of the brain were significantly higher than the median viral load in the cerebrum and cerebellum (Mann-Whitney; *P* < 0.05 for all comparisons).

When analyzing the amount of JEV RNA over time in each brain part , an increase of the mean viral load can be observed in the olfactory bulb and the thalamus upon intranasal inoculation with a peak at 10 dpi. Upon intradermal inoculation, this increase can be seen in the brain stem, cerebrum, olfactory bulb, and thalamus with a peak at 7 dpi. This increase over time seems indicative for a local virus replication. It is however only in the thalamus after intranasal (Krukall-Wallis; *P* = 0.05) and intradermal (Kruskal-Wallis; *P* = 0.01) inoculation that significant differences in the median viral load of positive animals over time were found.

Despite the differences in viral loads between parts of the brain and the indications for local replication in some tissues, the observed maximal viral RNA loads remained relatively low and were only equivalent to 10^3^–10^4^ TCID/g (pig A14 and A24 in the olfactory bulb and A17 and A18 in the thalamus after intranasal inoculation and B17 in the brain stem, B15 and B16 in the cerebrum, B14 in the olfactory bulb, and B16 in the thalamus after intradermal inoculation), with some exceptions of 10^5^ TCID50/g thalamus (B14, B15) after intradermal inoculation. These low viral loads detected by RT-qPCR were in line with the results of virus isolation and titration. JEV could only be isolated from the thalamus of pig B15 in which the highest viral load was detected at 7 dpi by RT-qPCR. The amount of the virus present was however below the detection limit for viral titration. All other brain samples remained negative in virus isolation.

**Fig. 4:**
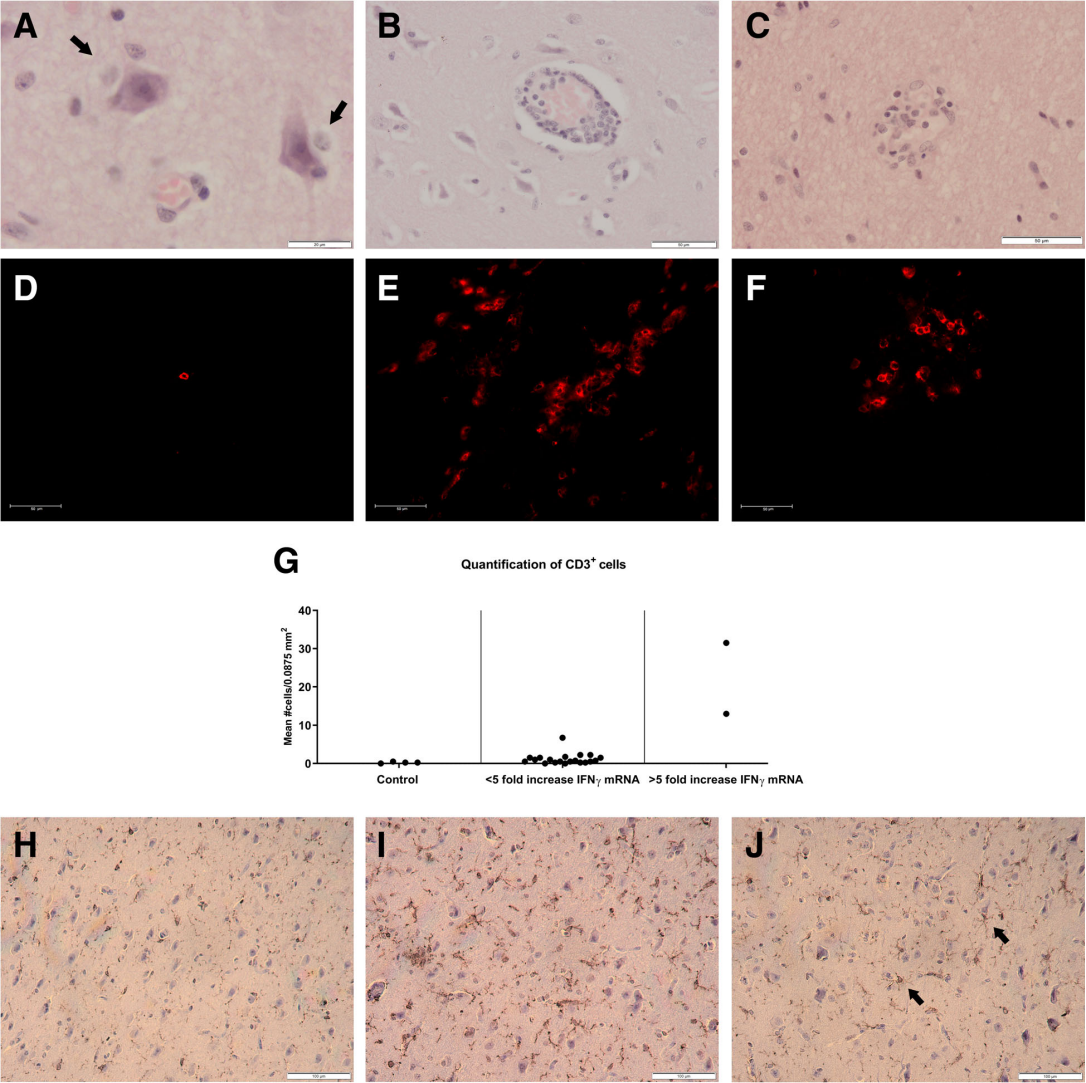
Histopathology, lymphocyte infiltration, and microglial activation in the cerebrum. Representative histopathologic lesions like perineuronal cuffing (**a**), perivascular cuffing (**b**), and noduli of microglial cells (**c**) observed in the cerebrum of animals B16 and B17 are shown. Representative pictures of infiltrating CD3+ T cells as visualized in the cerebrum of animals D2 (**d**), B15 (**e**), and B16 (**f**) are shown. Quantification of the number of CD3+ T cells in the cerebrum was done by counting their presence in 4 regions of 0.0875 mm^2^ under the fluorescence microscope of control pigs (D1, D2, D3, D8), pigs with a less than 5-fold increase in IFNy mRNA expression (A2, A3, A11, A13, A14, A15, A16, A17, A18, A22, A23, B2, B3, B11, B13, B14, B17, B22 B23), and pigs with more than 5-fold increase in IFNy expression (B15, B16). Each dot represents the mean of one animal (**g**). The activation status of microglial cells upon intradermal inoculation in the cerebrum was determined by quantification of IBA1 using the IHC profiler in ImageJ. Control pig D2 (**h**) shows a less intense IBA1 staining compared to pig B15 that showed an increased expression of pro-inflammatory cytokine mRNA (**i**). Even though scored as low positive, a more branched morphology of the microglial cells is seen in animal B16 as indicated by the arrows (**j**)

To further support the tropism of JEV to the CNS, histopathologic examinations were performed on formalin-fixed cerebrum samples collected after intradermal inoculation. Lesions supportive of non-suppurative encephalitis were readily found in animals in which JEV RNA was detected. Representative pictures of perineuronal and perivascular cuffing and foci of microglia and lymphocytes are shown in Fig. 4 a–c.

### mRNA expression in the CNS

Next, cytokine mRNA expression profiles were determined over time to analyze whether these could potentially provide an explanation for the observed differences in viral RNA loads between brain sections and possibly correlated with indications of local JEV replication in some of them (see above). Four cytokine groups (i.e., interferon and interferon-related cytokines, chemokines, inflammatory and anti-inflammatory cytokines, NLRP3 inflammasome-related proteins and cytokines) were studied in the cerebellum (no indications of JEV replication), cerebrum (indication of JEV replication only after intradermal inoculation), olfactory bulb, and thalamus (indications of JEV replication after inoculation via both routes).

**Fig. 5:**
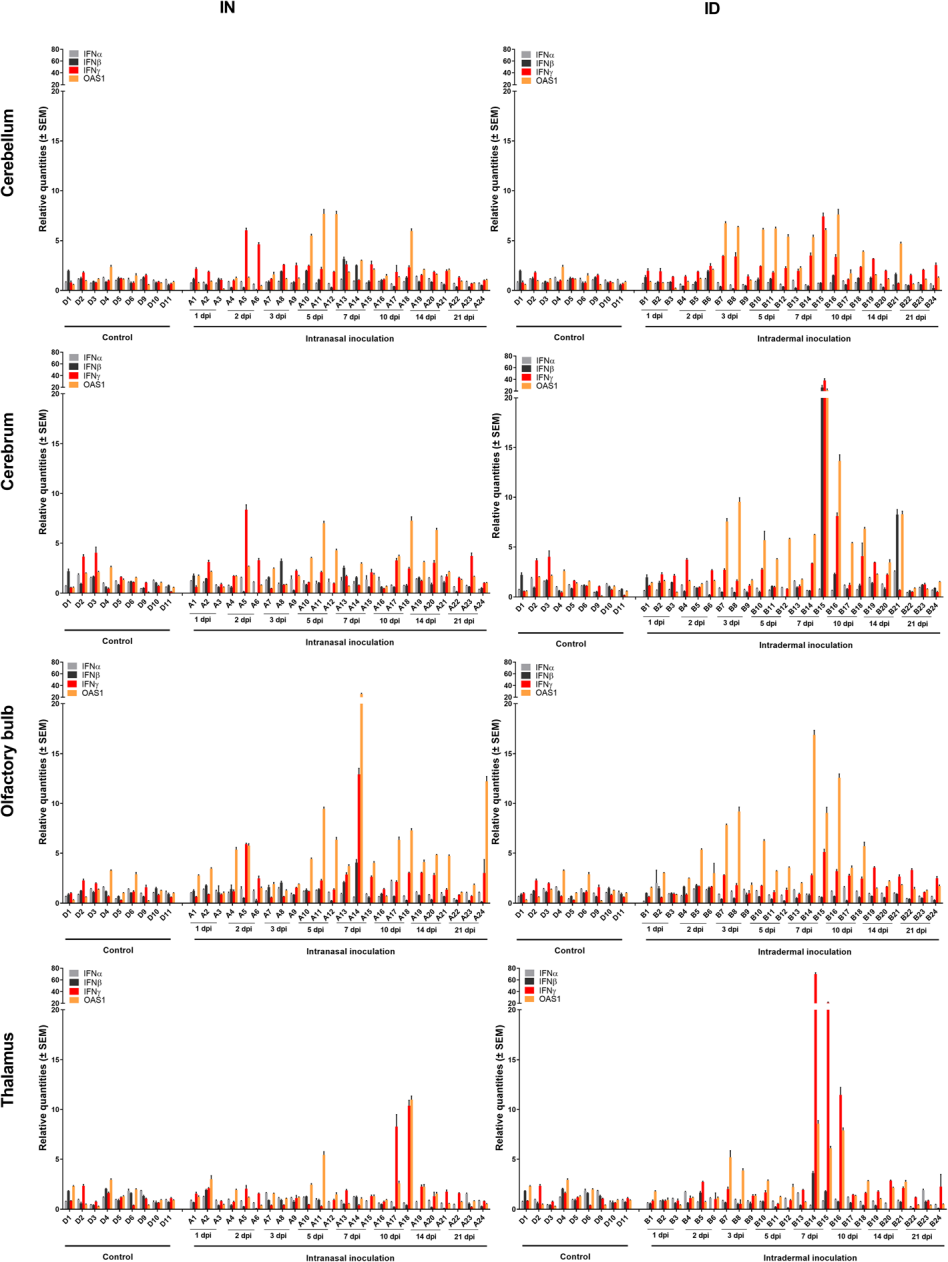
IFN-related antiviral immune response in the CNS. Antiviral immune response upon intranasal (IN) and intradermal (ID) inoculation of 9-week-old pigs with 10^5^ TCID_50_ JEV/animal. Fold-changes in IFNα, IFNβ, IFNγ, and OAS1 mRNA expression were determined by RT-qPCR and relative quantification in the cerebellum, cerebrum, olfactory bulb, and thalamus

Interestingly, no obvious changes in IFNα and IFNβ expression profiles were detected in any parts of the brain tested (Fig. 5). Nevertheless, independent of the inoculation route, the IFN-stimulated gene OAS1 was moderately (5- to 10-fold) upregulated in the cerebellum, cerebrum, and olfactory bulb of many pigs from the moment JEV RNA was detected (5 dpi intranasal inoculation and 3 dpi intradermal inoculation). The increase in OAS1 expression was less obvious in the thalamus. A more pronounced increase in OAS1 expression (10- to 20-fold) was seen especially in those animals with JEV RNA loads > 10^3^ TCID50/g, namely in the olfactory bulb of pig A14 and in the thalamus of pigs A17 and A18 after intranasal inoculation and in the cerebrum of pigs B15 and B16, in the olfactory bulb of B14, and in the thalamus of B14, B15, and B16 after intradermal inoculation. Also a strong increase in IFNγ expression (10- up to 50-fold) was observed in those same animals, except in the olfactory bulb of B14.

**Fig. 6:**
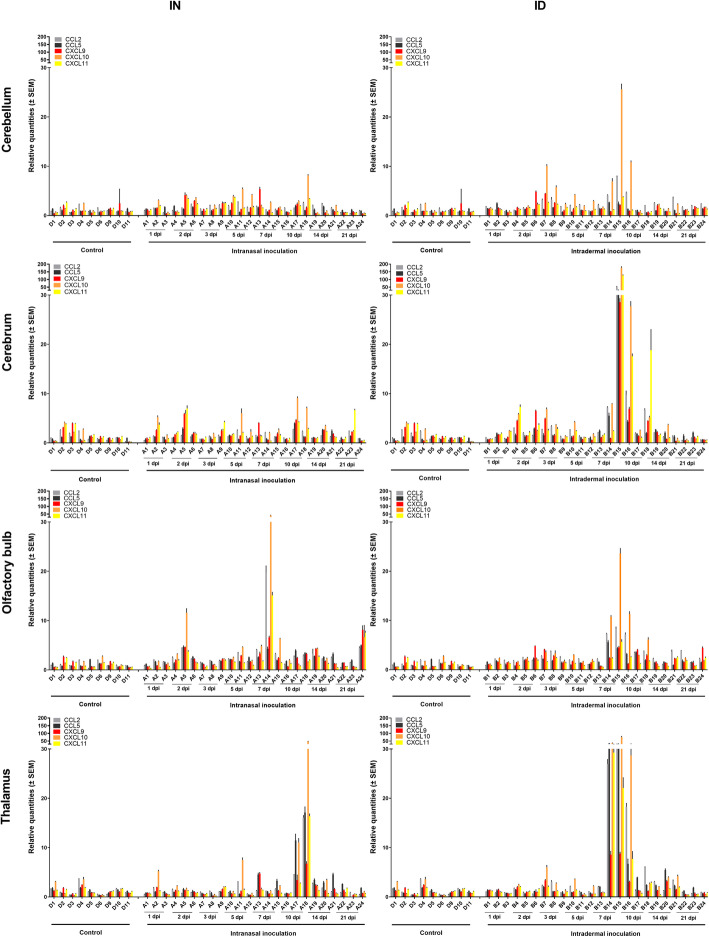
Chemokine mRNA expression in the CNS. Chemokine expression upon intranasal (IN) and intradermal (ID) inoculation of 9-week-old pigs with 10^5^ TCID_50_ JEV/animal. Fold-changes in CCL2, CCL5, CXCL9, CXCL10, and CXCL11 mRNA expression were determined by RT-qPCR and relative quantification in the cerebellum, cerebrum, olfactory bulb, and thalamus

No generalized increase in chemokine expression was observed in the different brain sections upon JEV infection (Fig. 6). Again, only those pigs in which the detected RNA levels suggested a local JEV replication showed a pronounced increase in chemokine expression. Upon intranasal inoculation, mRNA expression of chemokines raised at 10 dpi in the thalamus and 7 dpi in the olfactory bulb. The highest increase was observed for CXCL10 (IP10) (more than 50-fold) in pigs with high viral loads (in the thalamus of A17 and A18 and in the olfactory bulb of A14). CCL2 (MCP2) and CXCL11 showed a moderate increase in mRNA expression (15-fold) while no effect was seen for CXCL9 mRNA. A moderate 10- to 20-fold increase in CCL5 (RANTES) mRNA expression was found in the thalamus, while no change occurred in the olfactory bulb. Upon intradermal inoculation, the increase of chemokine mRNA expression seems to be more generalized and pronounced than after intranasal inoculation in those animals with increased JEV RNA loads. More than 50-fold increases in CCL2, CCL5, CXCL10, and CXCL11 levels were observed in the thalamus and cerebrum at 7 dpi and 10 dpi. Chemokine levels in the olfactory bulb were less pronounced.

**Fig. 7:**
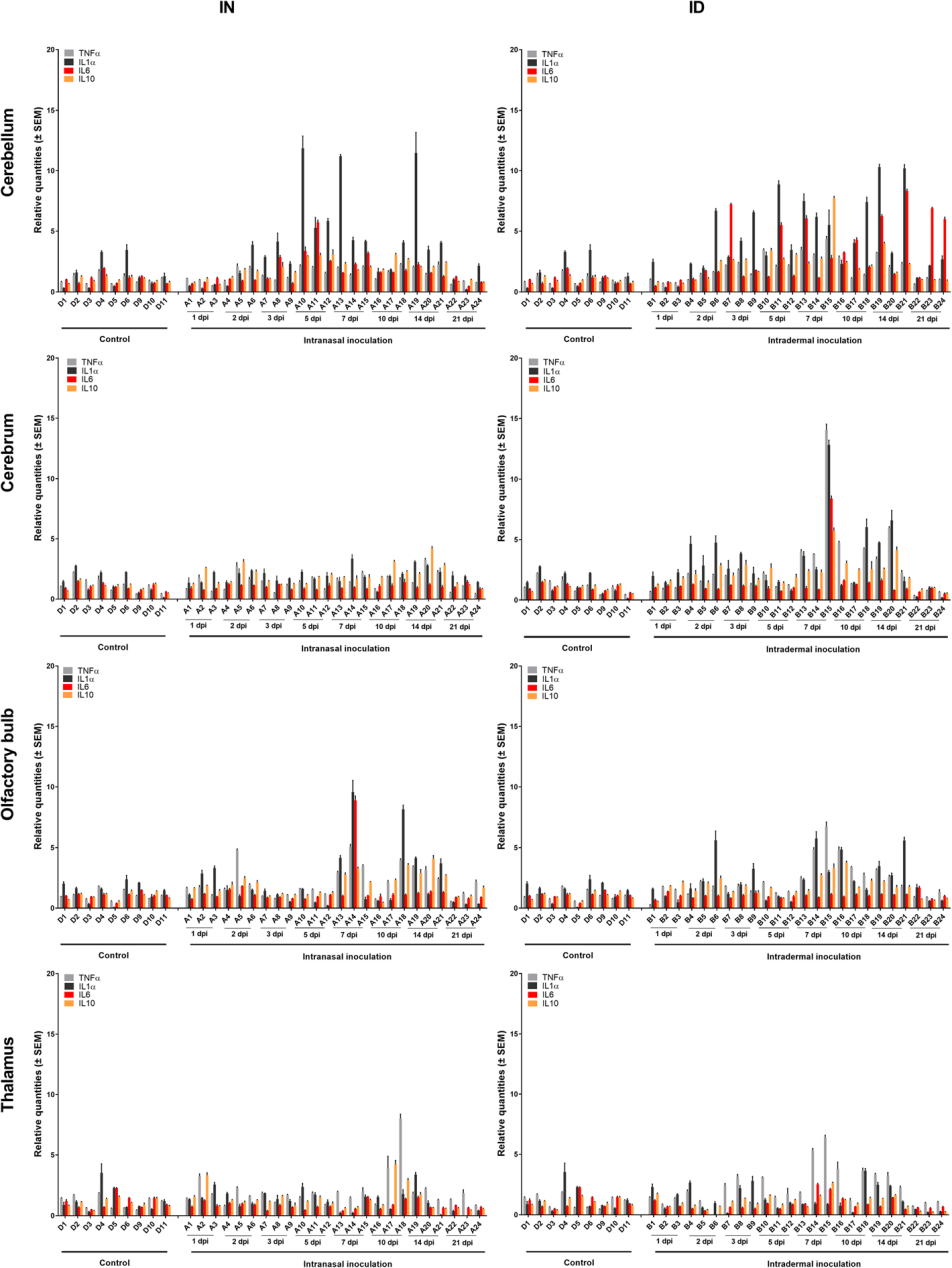
Inflammatory immune response in the CNS. Inflammatory immune response upon intranasal (IN) and intradermal (ID) inoculation of 9-week-old pigs with 10^5^ TCID_50_ JEV/animal. Fold-changes in TNFα, IL1α, IL6, and IL10 mRNA expression were determined by RT-qPCR and relative quantification in the cerebellum, cerebrum, olfactory bulb, and thalamus

Also no marked increase in mRNA expression of pro- and anti-inflammatory cytokine genes was observed in the brain of JEV infected pigs (Fig. 7). Some of the animals showing higher viral RNA loads had a limited 5- to 10-fold increase of TNFα and/or IL1α mRNA levels (e.g., animal B15 in the cerebrum), but overall, changes were minimal. No change in the anti-inflammatory cytokine IL10 was observed in any of the animals.

**Fig. 8:**
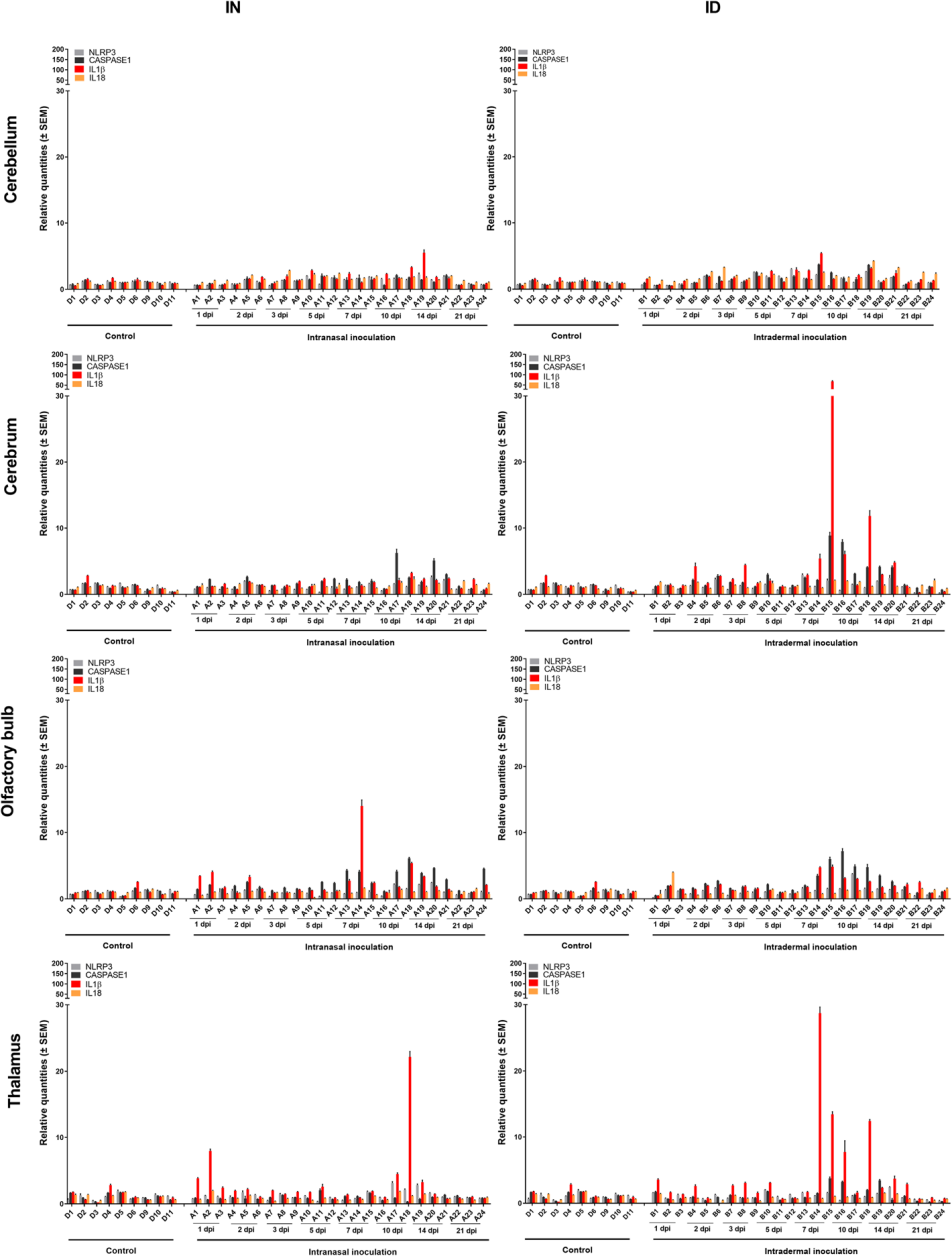
NLRP3 inflammasome-related immune response. Immune responses related to the NLRP3 inflammasome in the CNS upon intranasal (IN) and intradermal (ID) inoculation of 9-week-old pigs with 10^5^ TCID_50_ JEV/animal. Fold-changes in NLRP3, caspase 1, IL1β, and IL18 mRNA expression were determined by RT-qPCR and relative quantification in the cerebellum, cerebrum, olfactory bulb, and thalamus

Similarly, also the mRNA expression profile of several components and cytokines related to the NLRP3 inflammasome like NLRP3, Caspase 1, and IL18 remained unchanged independent of the route of inoculation in all tested tissues (Fig. 8). Only a 20- to 50-fold rise in IL1β mRNA expression in the cerebrum, olfactory bulb, and thalamus was observed in those pigs carrying the highest viral RNA loads.

### Infiltration of lymphocytes and activation of microglial cells

The detected increase in IFNγ mRNA expression in those animals with JEV RNA loads > 10^3^ TCID50/g (see above) raised the question whether this could be related to an infiltration of T cells. This was assessed by fluorescence IHC on frozen cerebrum sections upon intranasal and intradermal inoculation. Sections were stained for CD3 antigen to visualize T cells. Quantification showed that an infiltration of T cells coincides with the peak of IFNγ mRNA expression at 7 and 10 dpi in animals B15 and B16 (Fig. 4g). The highest number of infiltrating T cells is seen in animals with a more than 5-fold increase in IFNγ mRNA such as animal B15 followed by animal B16 (Fig. 4 e–g). Only a very limited number of T cells could be detected in the cerebrum of control animals and animals showing a less than 5-fold increase in IFNγ mRNA, independent of the route of inoculation (Fig. 4d, g).

Since only very few animals (e.g., B15 in the cerebrum) showed a limited increase in pro-inflammatory cytokine mRNA expression while most did not (see above), we performed IBA1 stainings on the cerebrum of some intradermal JEV-infected pigs to verify if differences in pro-inflammatory cytokine mRNA expression correlated with differences in the activation status of microglial cells in those animals. All samples were scored using the IHC profiler plugin. Animal B15 was the sole animal that scored positive (Fig. 4i). Control animals (D2, D3) (Fig. 4h) and JEV-infected animals without a rise in pro-inflammatory cytokines mRNA (B1, B14, B16, B17, B18) scored low positive although some samples showed a more dendritic profile (B16 and B18) (Fig. 4j). Thus, overall, the unchanged pro-inflammatory mRNA expression in most animals correlates with an absence of microglial activation.

## Discussion

Pigs are important amplification hosts of JEV and remain mostly asymptomatic upon infection. Mosquitoes can become infected during a blood meal on viremic pigs and spread the disease to other hosts. Recent data showed that also direct vector-independent JEV transmission between pigs is possible [6]. Despite their importance, only limited studies have addressed JEV neuropathogenesis and immune responses controlling JEV infections in the CNS of pigs. We therefore performed an in-depth study of these aspects of both intradermal and intranasal JEV-inoculated pigs, respectively, mimicking infection by mosquitoes and vector-free transmission.

Upon both intranasal and intradermal JEV inoculation of pigs, JEV was detected in nasal swabs. This is in line with other studies [6, 7, 19, 20] and supports the recent finding that vector-independent JEV transmission could occur. The number of JEV-excreting pigs and the amount of infectious virus recovered was however lower than in the other studies. This is probably due to differences in experimental set-up, like the use of different inoculation routes (intradermal, intranasal, intravenous) and/or differences in inoculation dose.

Previous studies reported viremia already after 1 dpi intravenous inoculation [6, 7]. In our experimental set-up, the peak viremia upon intradermal inoculation was reached after 3 dpi, while this was only after 5 dpi upon intranasal inoculation. This was the most important difference in JEV dissemination observed between both inoculation routes in our study and probably reflects differences in the route the virus has to travel before reaching the blood stream. Upon intradermal flavivirus infection, immune cells are recruited to the site of infection as a first line of defense. These cells may become infected and transport the virus into the lymphatic system [21, 22]. Our results showing that JEV RNA was already detected in the skin and draining lymph node after 1 and 2 dpi, respectively, before JEV became detectable in serum and leukocyte pellets, suggest that this might also occur upon JEV infection of pigs, but this needs to be confirmed by more detailed studies in the future. Next, the virus seems to migrate into the bloodstream where the peak viremia is reached at 3 dpi. The route followed by JEV to the blood circulation upon intranasal inoculation remains unclear. Potentially, a local replication occurs in the nasal mucosa followed by a replication in draining lymph nodes, but this currently remains speculative. Interestingly, however, relatively high viral loads of JEV RNA (≈10^4^–10^5^ TCID_50_) were detected in the prescapular lymph node from 5 dpi intranasal inoculation onwards. Since this is not the direct draining lymph node upon intranasal infection, it strengthens the suspicion that local lymph nodes may constitute an important place for viral replication upon infection. The observation that all visceral and lymphoid organs tested became JEV RNA-positive at the moment of peak viremia in both inoculation routes suggests a blood-borne spread of JEV throughout the body. The virus is however quickly removed from the blood by the developing humoral immune response, which also seems to limit viral replication in all those organs.

A key characteristic of many flaviviruses, including JEV, is their capacity to infect the CNS. In our study, JEV showed a particular tropism for the CNS without causing any neurologic symptoms, although signs of non-suppurative encephalitis were found during histopathology. It is nevertheless still not completely clear how flaviviruses enter the brain, but multiple strategies are probably exploited [23–25]. A first route to be considered is a hematogenous route of entry to the brain parenchyma, which can occur via direct infection of endothelial cells, a passage between disrupted endothelial tight junctions, or via hijacking of peripheral leukocytes migrating into the CNS. The hematogenous route has since a long time been described as a major entry route of JEV in the CNS of multiple hosts [24, 25]. Our observation that the moment of peak JEV detection in the serum and blood leukocytes during the short viremia coincides with the moment of first JEV detection in the cerebrum and cerebellum of most animals might support a JEV entry in the porcine brain via this route. As described for WNV and JEV in other hosts like humans and mice [25–28], infectious virus present in the serum could directly infect endothelial cells followed by transcellular release of the virus into the brain parenchyma or infected leukocytes could enter JEV in the CNS via a “Trojan Horse” mechanism. The third route via disruption of the BBB seems to be secondary to the entry into the CNS and is probably the result of aberrant cytokine production induced by the infection of microglia cells in the CNS [29, 30]. Next to a hematogenous entry, the detection of JEV RNA in the trigeminal ganglion, brain stem, thalamus, and olfactory bulb suggests that JEV can also use different peripheral nerve pathways to enter the brain. Indeed, flaviviruses can use axonal transport systems to travel transneurally into the CNS, thereby bypassing the BBB [31]. The low number of JEV RNA-positive TG samples with only low viral loads independent of the inoculation route however indicates that the entry route via the trigeminal nerve may not play an important role in JEV neuropathogenesis, not even upon intranasal inoculation. The same appears to be true for JEV entry in the brainstem via dorsal root ganglion neurons after intranasal inoculation.

Thus, although JEV seems to enter the CNS of pigs via the blood and several peripheral nerve routes, viral replication appears strongly hampered immediately upon entry in most CNS tissues of infected pigs as indicated by the presence of only low viral RNA loads over the time course of the experiment. This hampered viral replication in most animals correlated well with a generalized 5 to 10-fold increase in mRNA expression of the antiviral 2′,5′-oligoadenylate synthetase 1 (OAS1) gene observed at the moment of the first detection of JEV RNA in the brain at 3 and 5 dpi upon intradermal and intranasal inoculation, respectively. The OAS1 protein is a type I IFN-induced gene product and mediates its antiviral activity by activating RNase L, resulting in RNA degradation and inhibition of protein synthesis [32]. Our indications for an important role of this antiviral protein to limit JEV replication at an early stage of infection seem in line with in vitro results showing the capacity of OAS to inhibit JEV replication [33]. In this context, it was highly surprising that no obvious type I IFN response was observed in any of the studied brain tissues upon JEV infection. This is most probably due to the capacity of JEV to actively suppress IFN expression as was reported before [34–36]. In our experiment, the OAS gene expression could have been activated by a type I IFN-independent mechanism as has been reported for WNV infection [37].

The increased OAS1 expression alone however does not seem capable to control JEV replication in brain tissues of all infected pigs and especially not in the olfactory bulb and thalamus at 7 to 10 days post infection via both inoculation routes, since these samples showed coinciding high levels of JEV RNA and OAS1 expression. In fact, these tissues showed a significantly higher JEV RNA load than other brain tissues and indications for viral replication were found. Both thus seem to constitute JEV entry sites where virus replication could lead to clinical symptoms when replication is not constrained. JEV replication in the olfactory bulb has already been reported upon intranasal inoculation of 3-week-old pigs [8] and intravenous inoculation of 8-week-old pigs [7]. The most anticipated route to explain infection of the OB is via infection of olfactory neurons located in the nasal cavity mucosa after which the virus can reach the second-order neurons located in the olfactory bulb [23, 8]. In this respect, it was staggering to observe that after intranasal inoculation whereby JEV normally immediately infects the nasal mucosa [8], JEV RNA was only detected in the olfactory bulb at 5 dpi while this already occurred at 3 dpi intradermal inoculation, coinciding again with the moment of peak viremia for both inoculation routes. This suggests that in our 9-week-old pigs, JEV reaches the OB rather via the hematogenous than via the olfactory route, although this needs to be confirmed by more in-depth studies. A hematogenous entry of JEV in the OB would be in line with the observation that LaCrosse virus, another mosquito-borne virus, also spreads via the hematogenous route to the OB in mice and that capillaries in the OB could be hot spots for neuroinvasion [38]. Also the thalamus has already been shown to constitute an important tissue for JEV replication in the brain of mice [39], piglets [5], and macaques [40], and thalamic lesions are the most commonly described abnormality based on magnetic resonance imaging (MRI) of JEV patients [41–43]. Entry to the thalamus probably occurs via infection of the sensory neurons of the dorsal route ganglion which connects directly to the thalamus [23, 39]. Besides the significantly higher viral RNA loads in the olfactory bulb and thalamus, a few intradermal inoculated pigs also showed higher JEV RNA loads in the brain stem and cerebrum, and thus, it cannot be excluded that these brain tissues can also play a role in JEV replication in the CNS.

In those animals and brain tissues where the OAS1 response cannot control the JEV infection on itself, pigs still seem extremely efficient in halting JEV replication, likely via activation of other immune mechanisms, thereby preventing neurological symptoms. Indeed, together with a further increase in OAS1 mRNA expression, several chemokine genes were found to be activated, resulting in a more than 50-fold increased mRNA expression of some of them. Especially CXCL10 seems to be strongly induced in all brain tissues at the moment that increased viral RNA loads were observed, but also increased mRNA levels of CCL2, CCL5, and CXCL11 were detected, especially upon intradermal inoculation. Expression of these chemokines has been described to result in the attraction of a plethora of immune cells. Increased mRNA expression of CCL2 and CCL5 leads to an infiltration of macrophages and activated T cells [21, 44, 45], while CXCL9, CXCL10, and CXCL11 all have in common to bind to the chemokine receptor CXCR3, resulting in the attraction of monocytes, NK cells, dendritic cells, and probably the most important active T cells [30, 45]. Data from immunofluorescence stainings indicate that infiltrating T cells probably account for the strong increase in IFNy mRNA expression that could contribute to elimination of JEV-infected cells. This is in line with what has been reported for WNV infection in the brain [46, 47].

Surprisingly, the strongly increased expression of CXCL10 and other chemoattractant genes was not associated with a strong cellular pro-inflammatory response in any of the brain tissues tested, independent of the route of infection. Only in some specific tissues, a 5 to maximum 10-fold increase in TNFα, IL1α, and IL6 mRNA expression was found at 7 and 10 dpi. In the cerebrum, only the pig with such an increased pro-inflammatory response (B15) showed an increased activation status of its microglial cells. The absence of a pro-inflammatory response in most pigs is in sharp contrast to observations in humans, primates, and mice where an extensive increase in pro-inflammatory cytokine expression, including TNFα, IL6, IL8, and IL1α can be observed [9, 10, 13, 14, 45, 48], leading to neuronal cell death and correlating with clinical disease. The tight regulation or inhibition of the pro-inflammatory response in pigs might thus be part of the explanation as to why an infection with JEV in pigs is mostly subclinical. A hypothesis with regard to the underlying mechanism that prevents an inflammatory response might be found in the observation that neither NLRP3 nor caspase-1 was upregulated in JEV-infected porcine brain tissues. Both proteins are necessary components of the NLRP3 inflammasome, which in turn mediates the maturation of pro-inflammatory cytokines IL1β and IL18. In mice, it is known that neuronal inflammation and neuronal death associated with JEV pathogenesis strongly depends on the NLRP3 inflammasome and the resulting maturation of pro-IL1β and pro-Il18 into IL1β and Il18 [11, 12]. It has also been shown that inhibition of NLRP3 reduced the inflammatory pathology during chikungunya infection [49]. While no increase in IL18 mRNA expression was detected in our study, a strong increase in IL1β mRNA was however found in those tissues where higher JEV RNA loads were detected. Since NLRP3 is thought to exist at concentrations that are inadequate for initiating inflammasome activation under resting conditions [50] and no increase in NLRP3 and caspase-1 mRNA was detected, the resulting pro-IL1β probably remains present in its inactive precursor form that cannot exert its role as an important mediator of the inflammatory response.

Combined with the observed absence of a type I IFN response as discussed above, the absence of a pro-inflammatory immune response triggers the question whether JEV potentially might be capable to avoid recognition by PAMP receptors in immune cells of porcine brain tissues. Future research will have to shed light on this matter.

## Conclusions

In summary, both intranasal and intradermal JEV inoculation in pigs resulted in a disseminated JEV infection without induction of neurologic signs. Besides a delay of 2 days to reach the peak viremia, the overall virus spread via both routes is highly similar. Independent of the inoculation route, our results indicate that JEV entry in the CNS probably occurs via both hematogenous and neuronal pathways. JEV replication in the brain is presumably initially suppressed by innate antiviral responses such as OAS1. If this mechanism seems inefficient to halt JEV replication, a short but strong induction of chemokine gene expression, mainly by CXCL10 is observed. This is associated with an immune cell infiltration of activated T cells, most probably leading to a fast suppression of JEV replication by IFNγ expression. The chemokine response was not associated with the induction of a strong pro-inflammatory response, and most importantly, no upregulation was observed in genes involved in the NLRP3 inflammasome, which plays a key role in inflammatory neuropathology in other hosts. Our results show that a favorable outcome for JEV is associated with an adequate antiviral response and an attenuated pro-inflammatory immune response.

## Supplementary information

**Additional file 1.** Primer/probe sequences used for porcine reference gene and cytokine detection

**Additional file 2.** Body temperature of pigs upon intranasal (black), intradermal (red) or mock (grey) JEV inoculation of 9-week old pigs with 10^5^ TCID_50_/animal. Mean temperature and standard deviation of all pigs remaining at each time point are shown

**Additional file 3.** Amount of infectious JEV in serum determined by virus titrations upon intranasal (IN) and intradermal (ID) inoculation of 9-week old pigs with 10^5^ TCID_50_/animal

**Additional file 4.** JEV RNA loads in spleen, kidney, and liver determined by RT-qPCR upon intranasal (ID) and intradermal (ID) inoculation of 9-week old pigs with 10^5^ TCID_50_/animal. Each dot represents one animal

## Data Availability

The datasets supporting the conclusions of this article are included within the article and its additional files.

## Abbreviations

BBB: Blood-brain barrier
CNS: Central nervous system
dpi: Days post infection
JEV: Japanese encephalitis virus
OAS1: 2′,5′-Oligoadenylate synthetase 1
pi: Post infection
WNV: West Nile virus
IHC: Immunohistochemistry
