# Supplementary material for: Efficient control of Japanese encephalitis virus in the central nervous system of infected pigs occurs in the absence of a pronounced inflammatory immune response
Source: J Neuroinflammation. 2020 Oct 23;17:315. doi: 10.1186/s12974-020-01974-3 (PMC7585311; doi:10.1186/s12974-020-01974-3)
Supplement: Supplementary file 1 — Additional file 1. Primer/probe sequences used for porcine reference gene and cytokine detection [file 12974_2020_1974_MOESM1_ESM.docx]

Table 1. primer/probe sequences used for porcine reference gene and cytokine detection

|  |  | Primer or probe sequence (5’ – 3’) | | |  |
| --- | --- | --- | --- | --- | --- |
|  | **Description** | **Forward primer** | **Reverse primer** | **Probe** | **Source** |
| Reference genes |  |  |  |  |  |
| ACTB | β-actin | AGCGCAAGTACTCCGTGTG | CGGACTCGTCGTACTCCTGCTT | TCGCTGTCCACCTTCCAGCAGATGT | 16 |
| GADPH | glyceraldehyde-3-phosphte-dehydrogenase | ACATGGCCTCCAAGGAGTAAGA | GATCGAGTTGGGGCTGTGACT | CCACCAACCCCAGCAAGAGCACGC | 16 |
| PPIA | peptylpropyl isomerase A of cyclophilin A | TGCTTTCACAGAATAATTCCAGGATTTA | GACTTGCCACCAGTGCCATTA | TGCCAGGGTGGTGACTTCACACGCC | 16 |
| UBC | ubiquitin C | GCGCACCCTGTCTGACTACA | AGATCTGCATCCCACCTCTGA | AGTCCACCCTGCACCTGGTCCTCC | 16 |
| Targets of interest | |  |  |  |  |
| IFNα | Interferon alpha | CAGGTCCAGAAGGCTCAAG | ATCCAGTCCAGTGCAGAAC | AGGTCTGCTGGAGCATCTCATGC | 16 |
| IFNβ | Interferon beta | AGCAGATCTTCGGCATTCTC | GTCATCCATCTGCCCATCAA | TAGCACTGGCTGGAATGAAACCGT | 16 |
| IFNγ | Interferon gamma | CGATCCTAAAGGACTATTTTAATGCAA | TTTTGTCACTCTCCTCTTTCCAAT | ACCTCAGATGTACCTAATGGTGGACCTCTT | 16 |
| TNFα | Tumor necrosis factor alpha | AACCTCAGATAAGCCCGTCG | ACCACCAGCTGGTTGTCTTT | CCAATGCCCTCCTGGCCAACG | 16 |
| IL1α | Interleukin-1 alpha | GTGCTCAAAACGAAGACGAACC | CATATTGCCATGCTTTTCCCAGAA | TGCTGAAGGAGCTGCCTGAGACACCC | 16 |
| IL1β | Interleukin-1 beta | AAGTGCTGCACCCAAAACCT | CACTGCCACGATGACAGACA | TGGGAGCATCCAGCTGCAAATCTC | Primer3 |
| IL6 | Interleukin-6 | CTGGCAGAAAACAACCTGAACC | TGATTCTCATCAAGCAGGTCTCC | TGGCAGAAAAAGACGGATGC | 16 |
| IL10 | Interleukin-10 | CGGCGCTGTCATCAATTTCTG | CCCCTCTCTTGGAGCTTGCTA | AGGCACTCTTCACCTCCTCCACGGC | 16 |
| IL18 | Interleukin-18 | CGGAAGACAATTGCATCAGC | TCAAACACGGCTTGATGTCC | TGAAAACGATGAAGACCTGGAATCGG | Primer3 |
| CCL2 | C-C chemokine ligand 2 (MCP-1) | TCACCAGCAGCAAGTGTCCT | AGTCAGGCTTCAAGGCTTCG | CAAGACCATCGCGGGCAAGGA | Primer3 |
| CCL5 | C-C chemokine ligand 5 (RANTES) | AAGCCTTGAGCCTGAACTCG | GGAGCCCTGGGAGGTTTTAC | TTGTCCCAGCCAGCTTGGGAGG | Primer3 |
| CXCL9 | C-X-C chemokine ligand 9 | GCAGTGTTGCCTTGCTTTTG | TCCTTTGGCTGGTGTTGATG | CCTGACTCTGATTGGAGTTCAAGGAACCC | Primer3 |
| CXCL10 | C-X-C chemokine ligand 10 (IP10) | GGGCCGGAGAGAATCTACCT | TCGGGATGATGAACCATCTG | TGCTCCCACACTTGCAGGGCA | Primer3 |
| CXCL11 | C-X-C chemokine ligand 11 | GGTGTGAAGGGCATGGCTA | GACACCTTCCCGCTTTGAAC | TGTGCTACAACTATTCAAGGCTTCCCCA | Primer3 |
| CASP1 | Caspase-1 | GCCCGAGCTTTGATTGACTC | GGGGGTCGTCTCCACAAATA | TCGGAAAGGGCCCCAGGCAT | Primer3 |
| NLRP3 | NOD-, LRR- and pyrin domain-containing protein 3 | AGCAGGAGAGGGAGCATGAG | GCACAGGCTCAGAGTGTTGG | TGGCCCCGTGAGTTCCCTGA | Primer3 |
| OAS1 | 2'-5'-oligoadenylate synthetase 1 | GTTGTGAAGGGTGGCTCCTC | TGAGGAAGACGACGAGGTCA | ACCCTCAGGGGCCGATCAGATG | Primer3 |
