# Supplementary figures and images for: Efficient control of Japanese encephalitis virus in the central nervous system of infected pigs occurs in the absence of a pronounced inflammatory immune response
Source: J Neuroinflammation. 2020 Oct 23;17:315. doi: 10.1186/s12974-020-01974-3 (PMC7585311; doi:10.1186/s12974-020-01974-3)

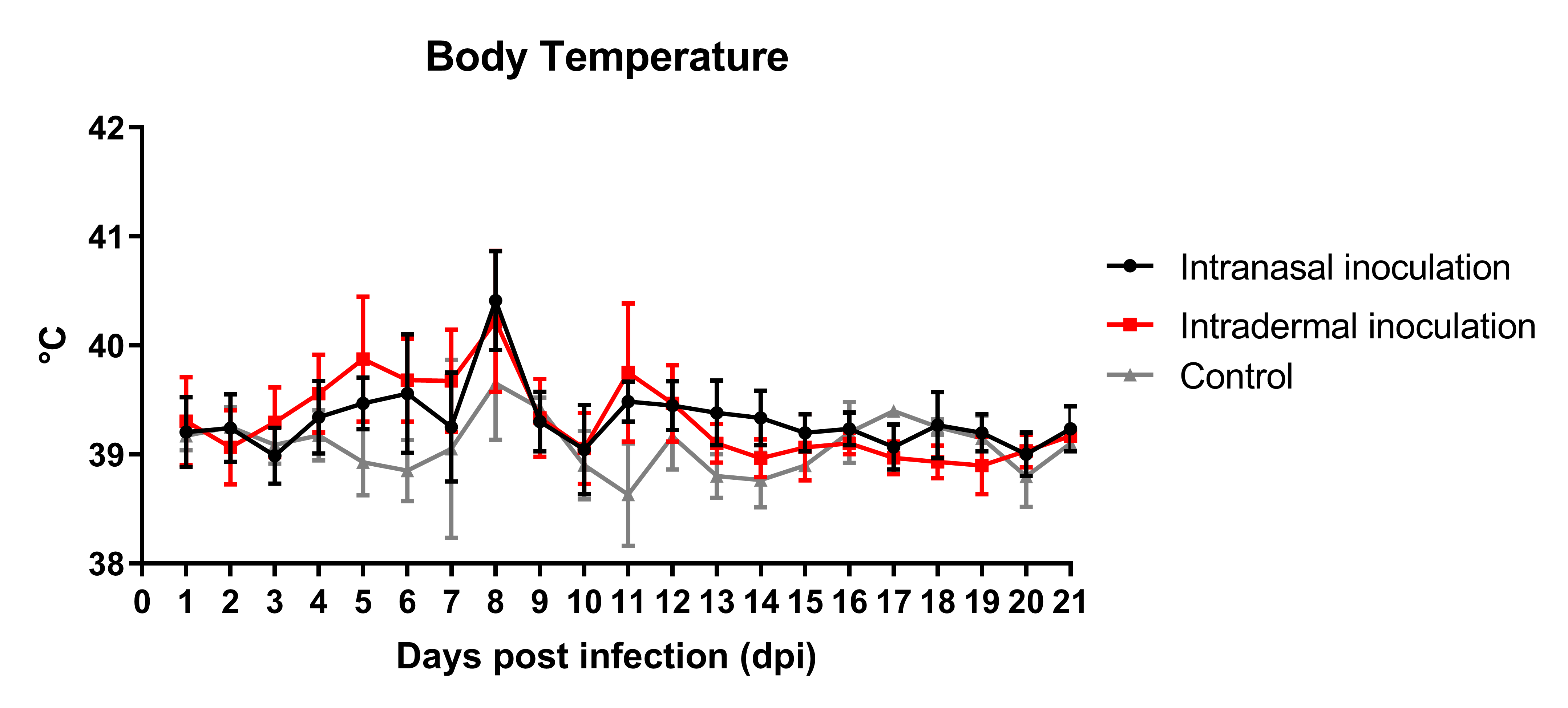

Supplement: Supplementary file 2 — Additional file 2. Body temperature of pigs upon intranasal (black), intradermal (red) or mock (grey) JEV inoculation of 9-week old pigs with 105 TCID50/animal. Mean temperature and standard deviation of all pigs remaining at each time point are shown [file 12974_2020_1974_MOESM2_ESM.tif]

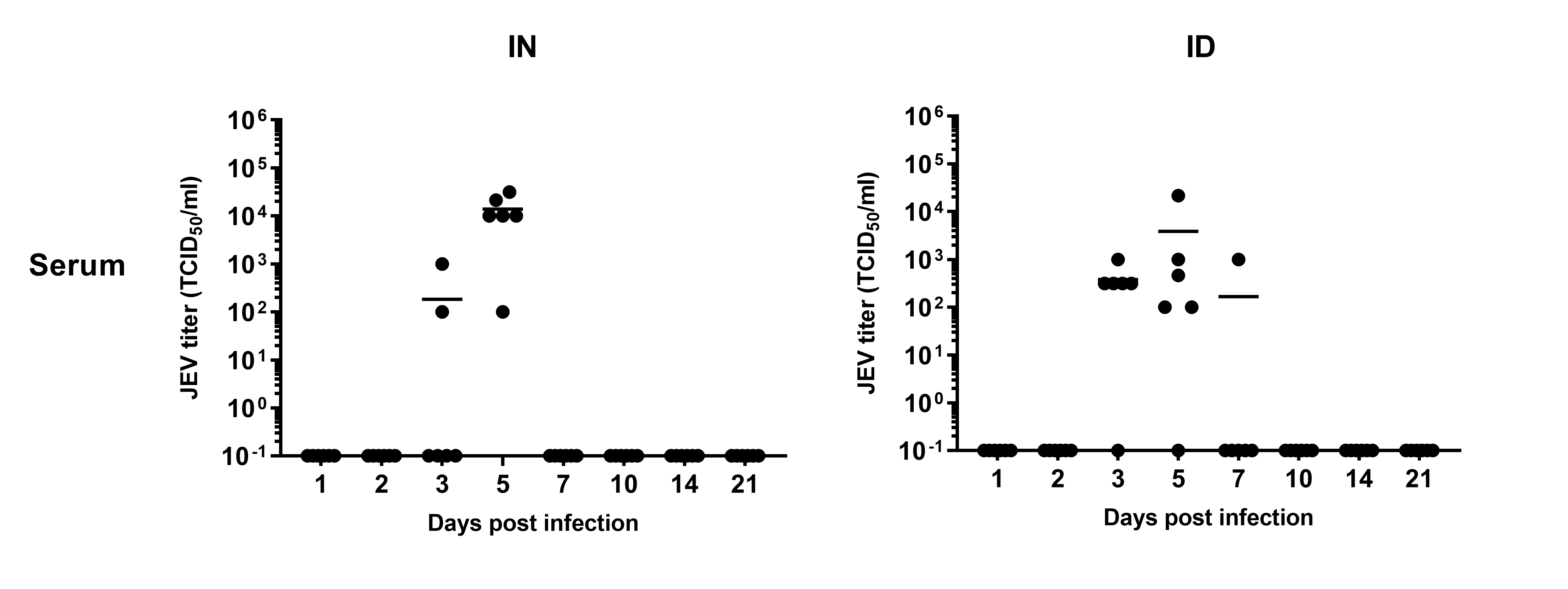

Supplement: Supplementary file 3 — Additional file 3. Amount of infectious JEV in serum determined by virus titrations upon intranasal (IN) and intradermal (ID) inoculation of 9-week old pigs with 105 TCID50/animal [file 12974_2020_1974_MOESM3_ESM.tif]

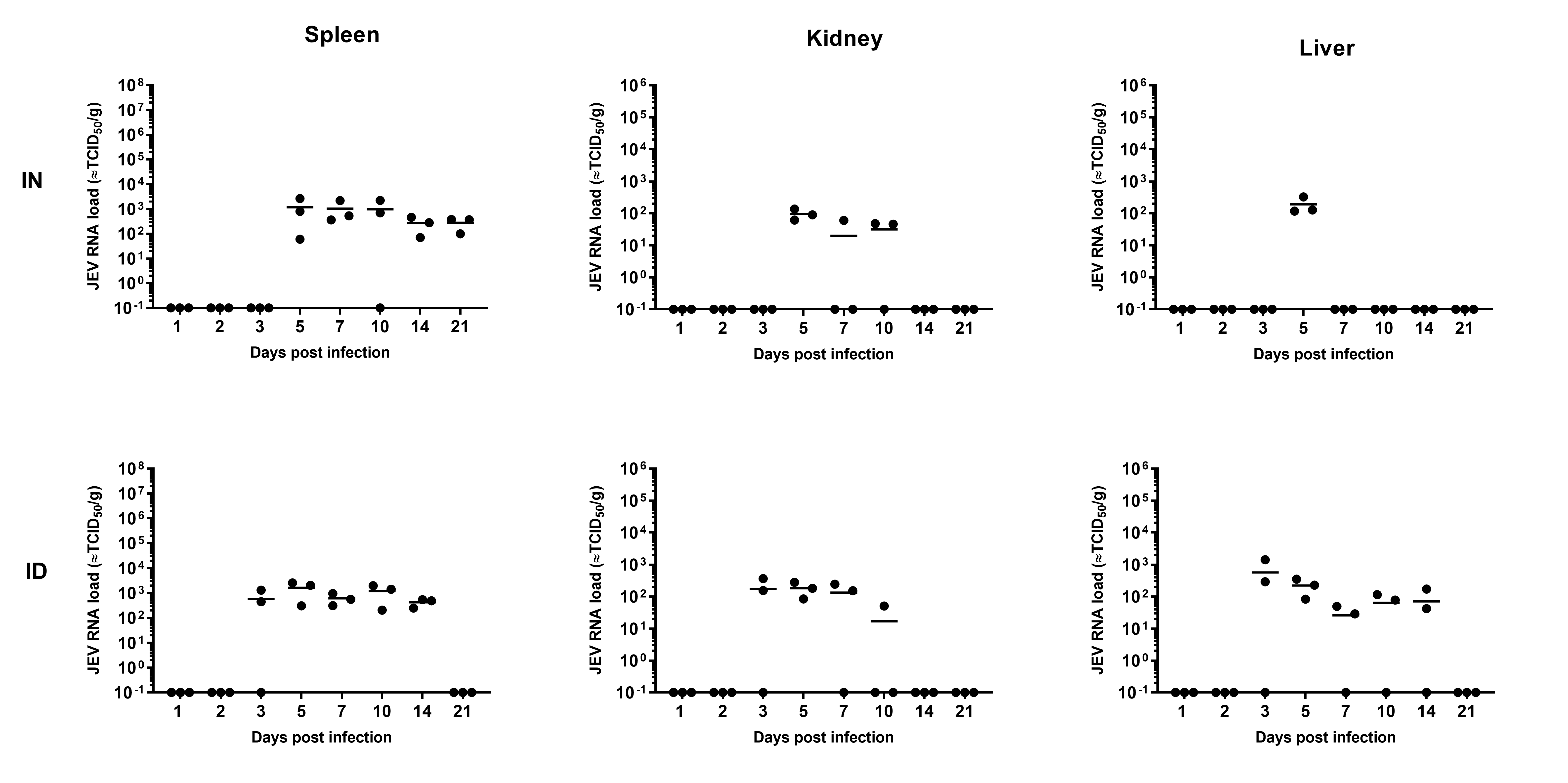

Supplement: Supplementary file 4 — Additional file 4. JEV RNA loads in spleen, kidney, and liver determined by RT-qPCR upon intranasal (ID) and intradermal (ID) inoculation of 9-week old pigs with 105 TCID50/animal. Each dot represents one animal [file 12974_2020_1974_MOESM4_ESM.tif]
